# Reliability and validity of the 6-item Headache Impact Test in chronic migraine from the PROMISE-2 study

**DOI:** 10.1007/s11136-020-02668-2

**Published:** 2020-10-20

**Authors:** Carrie R. Houts, James S. McGinley, R. J. Wirth, Roger Cady, Richard B. Lipton

**Affiliations:** 1 Vector Psychometric Group, LLC, Chapel Hill, NC USA; 2 Lundbeck Seattle BioPharmaceuticals, Inc, Bothell, WA USA; 3 Department of Neurology, Albert Einstein College of Medicine, Bronx, NY USA

**Keywords:** HIT-6, Construct validity, Chronic migraine, Item response theory, PROMISE-2

## Abstract

**Purpose:**

We examined the reliability and validity of the 6-item Headache Impact Test (HIT-6) specifically on patients with chronic migraine (CM) from the PROMISE-2 clinical trial.

**Methods:**

The conceptual framework of HIT-6 was evaluated using baseline data from the PROMISE-2 study (NCT02974153; *N* = 1072). A unidimensional graded response model within the item response theory (IRT) framework was used to evaluate model fit and item characteristics. Using baseline and week 12 data, convergent and discriminant validity of the HIT-6 was evaluated by correlation coefficients. Sensitivity to change was assessed by evaluating correlations between HIT-6 scores and change scores for other established reference measures. All examined correlations were specified a priori with respect to direction and magnitude. Known-groups analyses were anchored using Patient Global Impression of Change and monthly headache days at week 12.

**Results:**

A unidimensional model fit the data well, supporting that the 6 items measure a single construct. All item slopes and thresholds were within acceptable ranges. In both the validity and sensitivity to change analyses, all observed correlations conformed to directional expectations, and most conformed to magnitude expectations. Known-groups analyses demonstrated that the HIT-6 total score can distinguish between clinically meaningful CM subgroups.

**Conclusion:**

The HIT-6 was successfully calibrated using IRT with data from PROMISE-2. Results from these analyses were generally consistent with previous literature and provided supportive evidence that the HIT-6 is well suited for measuring the impact of headache and migraine in the CM population.

## Introduction

Chronic migraine (CM) is a common neurological disorder defined as having 15 or more headache days per month for more than 3 months with at least 8 days per month having features of migraine with or without aura [1]. Previous research has shown associations between CM and increased headache impact and disability as well as decreased health-related quality of life (HRQoL) [2–5]. Migraine is associated with increased familial burden and elevated direct and indirect medical costs [6–9], as well as increased occurrence of fatigue, irritability, headache pain severity, and comorbidities [10–12].

Preventive treatments for migraine are intended to decrease the frequency and impact of migraine attacks. A typical endpoint in migraine prevention trials is the mean change in monthly migraine days (MMDs) relative to pre-treatment baseline levels. Over the past two decades, however, the importance of using patient-reported outcome measures (PROMs) as secondary measures to better characterize the patient experience and potential treatment benefits has been recognized.

Many PROMs have been included in migraine prevention studies. One of these, the 6-item Headache Impact Test (HIT-6) [13], recommended by the American Headache Society [14], is intended to measure the impact of headache on daily life, with higher scores reflecting greater migraine impact [16]. The HIT-6 measures headache-related impact on six items, including severe headache pain, limitations to usual daily activities, the wish to lie down, fatigue, negative affect, and limitations to concentration. The items of the HIT-6 were selected from a large headache-related item bank [15] developed based on item response theory (IRT) parameters.

A substantial body of literature supports the HIT-6 as a precise and reliable PROM for assessing the impact of headache in the general headache population, as well as in patients with migraine [12, 13, 16–19]. However, much of the previous research has evaluated the broad headache population, and there is limited work specifically focused on use of the HIT-6 in CM, which is a particularly debilitating condition with features unique from other headache and migraine disorders.

The objective of the current research was to expand the existing knowledge base regarding the psychometric properties and evidence for validity of the HIT-6 in the CM population using data from a large clinical trial. Analyses were conducted to examine the model fit and individual item performance of the HIT-6 items using IRT, as well as to examine the internal consistency and test–retest reliability of the HIT-6 summed scores in a CM-specific sample. In addition, we performed analyses to examine convergent and discriminant validity and to evaluate the ability of the HIT-6 total score to distinguish between known groups and to demonstrate change.

## Methods

### Data source

The PRevention Of Migraine via Intravenous ALD403 Safety and Efficacy‒2 (PROMISE-2) study (ClinicalTrials.gov Identifier: NCT02974153) was a phase 3, randomized, double-blind, placebo-controlled trial evaluating the safety and efficacy of eptinezumab for the prevention of CM [20]. Eligible patients (*N* = 1072), with a diagnosis of CM per the International Classification of Headache Disorders third edition (beta) [21], were randomized to receive eptinezumab 100 or 300 mg, or placebo, administered by 30-min intravenous infusion once every 12 weeks.

Study approval for PROMISE-2 was provided by the independent ethics committee or institutional review board at each study site. The research was conducted in accordance with current Good Clinical Practice, the principles of the Declaration of Helsinki, and local regulatory requirements. Each enrollee provided written informed consent prior to their participation.

### Study measures

The current analyses used all available HIT-6 data from the PROMISE-2 study, pooling active treatment and placebo groups. For the reliability analyses, all data from the screening and baseline visits of those patients who passed screening and were accepted into the trial were evaluated. For the validity analyses, all data on measures and variables of interest at baseline and week 12 time points were evaluated.

The HIT-6 [13] measures the impact and effect of headache on the ability to function normally in daily life, and consists of six questions, each with five verbal response categories. Per the HIT-6 User’s Manual [22], the following values are used to score responses: never = 6, rarely = 8, sometimes = 10, very often = 11, and always = 13; these category weights were selected so that HIT-6 summed scores would correspond as closely as possible to scores from response pattern-based IRT scoring [13]. The total score was obtained by summing the responses to all six items using item weights just specified. Scores ≥ 60 were indicative of severe life impact, 56–59 of substantial life impact, 50–55 of some life impact, and ≤ 49 of little to no life impact. For the reported item-level analyses (item-level descriptive statistics, latent variable modeling, classical test theory analyses), the ordinal HIT-6 responses were coded as: never = 1, rarely = 2, sometimes = 3, very often = 4, and always = 5.

Baseline MMDs and monthly headache days (MHDs) were the number of migraine or headache days, respectively, reported during the 28-day screening period.

The Patient Global Impression of Change (PGIC) [23] was a single question concerning the patient’s impression of the change in their disease status since the start of the study. Verbal responses were scored on a seven-category scale (from “very much improved” to “very much worse”). The Short-Form Health Survey (SF-36 v2.0) [24] is a widely used, 36-question assessment measuring 8 domains of HRQoL (physical functioning, physical role functioning, emotional role functioning, vitality, mental health, social functioning, bodily pain, and general health) over the previous 4 weeks. Domain scores are created from between 2 to 10 items, depending on domain, and all have been found to exhibit suitable reliability in a wide variety of populations [25, 26]. The current analyses focused on the domains of bodily pain, physical role functioning, and emotional role functioning, in which higher scores indicate better functioning/health. The EuroQol five-dimension, five-level scale (EQ-5D-5L) [27] consists of five dimensions/items (scored using integer values ranging from 1 = “no problems” to 5 = “extreme problems”) and a visual analog scale (VAS; scored from 0 = “the worst health you can imagine” to 100 = “the best health you can imagine”). The current analyses focused on the individual item responses related to usual activities, pain/discomfort, and mobility dimensions.

### Data handling

All analyses were performed by pooling treatment arms and sites using all available data. Data management, descriptive summaries, and statistical tests were conducted using SAS software, version 9.4 (Cary, NC, USA).

No specific rules for missing item-level data on the HIT-6 are contained within the User’s Manual [22]. To be conservative, no imputation for missing data was used in these analyses, meaning that HIT-6 total scores were not to be calculated for any observations with missing item responses. No corrections were made for multiple testing to control Type I errors; the broader purpose of any presented *p* value was to help describe general patterns of effects regarding the HIT-6 scores.

### Analytic plan

#### Item-level descriptive statistics

Descriptive statistics and an observed frequency table for each of the HIT-6 items at the baseline assessment were examined for floor effect, ceiling effect, and missing data issues. The floor and ceiling effects were evaluated by looking at the percentages of responses in the lower and upper extreme response categories (i.e., “never” and “always”). Prior to IRT analyses, HIT-6 item responses were collapsed, if necessary, to obtain a minimum of five observed responses in each analyzed response category, to ensure sufficient observations in each category for accurate parameter estimation.

#### Unidimensional model fit

In consideration of the extensive psychometric work used to develop the HIT item bank and select items for the HIT-6, exploratory latent variable models were deemed unnecessary to assess the degree to which the HIT-6 items conformed to the theoretical model underlying them; however, a unidimensional IRT model was fit to the baseline HIT-6 data in flexMIRT 3.5 [28]. Given the ordered categorical response scale for all items, the IRT item model used was the graded response model [29]. Maximum marginal likelihood via the Bock-Aitkin expectation–maximization algorithm [30] was used to estimate IRT parameters; as this is a full-information estimation method [31], all observations (including those with item-level missing responses) were to be included in the analyses. Standard errors (SEs) were calculated via the supplemented expectation–maximization algorithm [32]. The fit of each model was evaluated using the Tucker-Lewis index (TLI) [33], and the limited information M_2_-based root mean square error of approximation (RMSEA) [34, 35], using customary cut-offs for adequate fit of ≥ 0.95 for the TLI [36] and < 0.08 for the RMSEA [37]. Item-level fit was evaluated using the summed-score-based item-fit diagnostic S-X^2^ [38, 39].

### Internal consistency reliability

To assess internal consistency/reliability, classical test theory analyses (i.e., item-total correlations, coefficient alpha, and alpha with item removed) were computed for the HIT-6 using baseline data, along with the IRT-based reliability plot and the IRT-based marginal reliability estimate. For the interested reader, Edwards [40] provides a general introduction to IRT, including how the concept of reliability in IRT varies from traditional, single-number summary values.

Coefficient alpha was calculated using two methodologies in recognition of the ordinal scale of the HIT-6 item responses: (i) based on Pearson correlations (traditional approach) using SAS software and (ii) based on polychoric correlations (modified approach) using R v3.4.3 [41]. Consistent with the HIT-6 manual [22] and the assumptions underlying coefficient alpha, only HIT-6 observations with complete item responses were used for the internal consistency reliability analyses. A minimum value of 0.70 demonstrated satisfactory reliability in evaluating both the IRT marginal reliability and coefficient alpha values [42].

#### Test–retest reliability

Test–retest correlations were calculated from screening to baseline for the HIT-6 total summed scores via uncorrected Pearson correlations and intraclass correlations (ICCs) from a two-way mixed-effect model with absolute agreement for single measures [43]. It was expected that patients would be relatively stable as both assessments occurred prior to study treatment; therefore, an anchor variable to define stability was not needed.

#### Convergent and discriminant validity

When correlations were examined between HIT-6 total scores and another continuous variable, Pearson correlations were used. When examined in relationship with a categorical/ordinal variable, Spearman correlations were used. All planned correlations were pre-specified with respect to expected direction and strength/effect size by a team comprising migraine experts and statistical methodologists [44]. With regard to effect size, a correlation of 0.1 indicated a small effect, 0.3 indicated a moderate effect, and 0.5 indicated a large effect size.

Trial eligibility criteria tend to create a homogeneous sample at the beginning of a study [45] and statistical theory tells us that having reduced variability within a sample can lead to artificially lowered correlations [46]. Since variability in all measures tends to increase over the course of the trial (due to treatment effects), we also examined a subset of the convergent/discriminant correlations at week 12, when greater heterogeneity in variables was expected and correlations would be unattenuated.

#### Known-groups validity

Distinct groups were created using the week 12 data. The “improved” group comprised those patients with PGIC item responses of “very much improved” and “much improved”. The “not improved” group contained those patients with responses of “minimally improved”, “no change”, “minimally worse”, and “much worse”. Similar analyses were conducted using groups defined by headache frequency during weeks 9‒12. Patients who reported ≥ 15 headache days during the 4-week period were classified as “chronic” (consistent with clinical practice), while patients with < 15 headache days over the same period were classified as “non-chronic”. All group differences were examined against typical Cohen’s *d* criteria where 0.2 indicated a small effect, 0.5 a moderate effect, and 0.8 or greater a large effect size [47].

#### Sensitivity to change

Change scores for HIT-6 total scores and individual HIT-6 items were correlated with change scores for other validation measures. Change on any variable of interest was defined from baseline to week 12; week 12 MMD and MHD values were defined as the number of migraine or headache days, respectively, reported between weeks 9 and 12 to match the 4-week recall period of the HIT-6 scores.

## Results

### Missing data

Analyses were based on all available data and missing data were extremely rare. Complete observations for the HIT-6 items were present as both baseline and Week 12 (i.e., if a patient was presented the HIT-6, all items were answered). The retention rate over the course of the trial on the primary efficacy variables was excellent (96% of patients; *n* = 1024). With respect to reference measures, only one patient did not provide responses for the PGIC and EQ-5D-5L (*n* = 1023 for these analyses) at Week 12.

### Sample and item-level descriptive statistics

Summary values for select demographic variables are provided in Table 1, pooling across the treatment groups. Mean age was 40.5 years, and patients were primarily female (88.2%), white (91.0%), and not Hispanic or Latino (92.0%).

**Table 1:** Baseline demographic information for the PROMISE-2 patients, pooling active treatment and placebo groups

	Pooled population *N* = 1072
Age (years), mean (SD)	40.5 (11.2)
Gender, *n* (%)	
Female	946 (88.2%)
Male	126 (11.8%)
Race, *n* (%)	
White	975 (91.0%)
Black or African American	82 (7.6%)
Multiple	7 (0.7%)
American Indian or Alaska native	3 (0.3%)
Asian	3 (0.3%)
Native Hawaiian or Other Pacific Islander	1 (0.1%)
Other	1 (0.1%)
Ethnicity, *n* (%)	
Hispanic	86 (8.0%)
Not Hispanic or Latino	986 (92.0%)

*SD* standard deviation

Individual HIT-6 item summaries for both the raw and the recoded/collapsed responses at baseline are provided in Table 2. To achieve the minimum of five observed responses in each analyzed response category, the responses for “never” and “rarely” were combined/recoded for items 1–3, creating a category of “never/rarely” that was used in the model fit analyses. As expected, patients in PROMISE-2 had HIT-6 total scores in the severe range at baseline [20]. Patients generally responded toward the higher end of the response scale for all items, as evidenced by both the provided item means and frequencies per category. Treating the item responses as numeric values (on a 1–5 response scale as noted previously), the mean item response values ranged from 3.52 to 4.24. For all six items, the most frequently used response category was “very often.” In terms of lived experience of the PROMISE-2 patients, these values indicate that patients’ lives are likely profoundly impacted by CM.

**Table 2:** Observed HIT-6 item response frequencies at PROMISE-2 baseline

Item	Item content	Descriptives		Response categories				
Raw/Not collapsed								
		Mean	SD	Never	Rarely (%)	Sometimes (%)	Very often (%)	Always (%)
1	Severe pain	3.68	0.63	0	28 (2.6)	350 (32.6)	626 (58.4)	68 (6.3)
2	Limits daily activities	3.64	0.73	3 (0.3%)	51 (4.8)	377 (35.2)	535 (49.9)	106 (9.9)
3	Lie down	4.24	0.78	4 (0.4%)	22 (2.1)	135 (12.6)	461 (43.0)	450 (42.0)
4	Too tired	3.52	0.80	11 (1.0%)	94 (8.8)	379 (35.4)	506 (47.2)	82 (7.6)
5	Felt fed up or irritated	3.69	0.92	18 (1.7%)	86 (8.0)	304 (28.4)	461 (43.0)	203 (18.9)
6	Limits concentration	3.63	0.80	9 (0.8%)	74 (6.9)	334 (31.2)	540 (50.4)	115 (10.7%)

After collapsing								
		Mean	SD	Never/Rarely (%)		Sometimes (%)	Very often (%)	Always
1	Severe pain	2.68	0.63	28 (2.6)		350 (32.6)	626 (58.4)	68 (6.3)
2	Limits daily activities	2.65	0.73	54 (5.0)		377 (35.2)	535 (49.9)	106 (9.9)
3	Lie down	3.25	0.76	26 (2.4)		135 (12.6)	461 (43.0)	450 (42.0)

				Never (%)	Rarely (%)	Sometimes (%)	Very often (%)	Always (%)
4	Too tired	3.52	0.80	11 (1.0)	94 (8.8)	379 (35.4)	506 (47.2)	82 (7.6)
5	Felt fed up or irritated	3.69	0.92	18 (1.7)	86 (8.0)	304 (28.4)	461 (43.0)	203 (18.9)
6	Limits concentration	3.63	0.80	9 (0.8)	74 (6.9)	334 (31.2)	540 (50.4)	115 (10.7)

The mean and standard deviation (SD) for the HIT-6 items are provided for descriptive purposes only and were calculated by scoring the ordinal responses with integer scores from 1 = never to 5 = always. *HIT-6* 6-item Headache Impact Test. *N* = 1072

Descriptive summary statistics for scores on all relevant and available PROMs at baseline and week 12 are reported in Table 3. From baseline to week 12, patients reported fewer MMDs and better outcomes.

**Table 3:** Descriptive statistics of the HIT-6 total score and reference variables by time point

	Baseline		Week 12	
*Continuous scores*	*n*	Mean (SD)	*n*	Mean (SD)
HIT-6 total score	1072	64.95 (5.13)	1024	58.61 (7.85)
MMDs (past 4 weeks)	1072	16.14 (4.64)	1024	8.71 (7.26)
MHDs (past 4 weeks)	1072	20.47 (3.10)	1024	12.04 (7.40)
EQ-5D-5L VAS	1071	75.69 (17.30)	1025	79.80 (16.30)
SF-36 physical role functioning	1072	42.01 (9.36)	1024	46.77 (8.60)
SF-36 bodily pain	1072	39.94 (9.55)	1024	45.05 (9.34)
SF-36 emotional role functioning	1072	46.72 (11.0)	1024	48.67 (9.28)

*Categorical/ordinal scores*	*n*	%	*n*	%
PGIC				
Very much improved	‒	‒	165	16.1
Much improved	‒	‒	360	35.2
Minimally improved	‒	‒	235	22.9
No change	‒	‒	231	22.6
Minimally worse	‒	‒	29	2.8
Much worse	‒	‒	3	0.3
Very much worse	‒	‒	1	0.1
EQ-5D-5L usual activities				
No problems	626	58.4	701	68.4
Slight problems	281	26.2	228	22.2
Moderate problems	129	12.0	81	7.9
Severe problems	31	2.9	14	1.4
Extreme problems	5	0.5	1	0.1
EQ-5D-5L pain/discomfort				
No problems	903	84.2	876	85.5
Slight problems	124	11.6	119	11.6
Moderate problems	38	3.5	27	2.6
Severe problems	6	0.6	3	0.3
Extreme problems	1	0.1	0	0.0
EQ-5D-5L mobility				
No problems	294	27.4	373	36.4
Slight problems	388	36.2	387	37.8
Moderate problems	259	24.2	209	20.4
Severe problems	109	10.2	48	4.7
Extreme problems	22	2.1	8	0.8

MMDs were based on values obtained from the missing data/weighting algorithm outlined in the PROMISE-2 study protocol. Week 12 summary values include only those patients with an observed HIT-6 total score at week 12

*EQ-5D-5L* EuroQol five-dimension, five-level scale, *HIT-6* 6-item Headache Impact Test, *MHDs* monthly headache days, *MMDs* monthly migraine days, *PGIC* Patient Global Impression of Change, *SD* standard deviation, *SF-36* Short-Form Health Survey (v2.0), *VAS* visual analog scale

### Unidimensional model fit

The single construct thought to underlie the HIT-6 was evaluated as a 6-item, unidimensional, graded response model [29]. The overall fit of this unidimensional model was acceptable (limited information M_2_-based RMSEA = 0.04; TLI = 0.95) and item slopes and thresholds all had reasonable values (Table 4). The fifth item (felt fed up or irritated) exhibited statistically significant (non-adjusted) misfit; S-X^2^(42) = 89.4, *p* < 0.001. However, given the acceptable fit of the overall model, the established nature of the HIT-6, and the clinical relevance of the item content [48], the decision was made to retain item 5.

**Table 4:** Item response theory unidimensional model of the HIT-6 at PROMISE-2 baseline

Item	Item content	*a* (SE)	*b*_1_ (SE)	*b*_2_ (SE)	*b*_3_ (SE)	*b*_4_ (SE)
1	Severe pain	1.12 (0.09)	‒3.72 (0.29)	‒0.68 (0.08)	2.86 (0.21)	
2	Limits daily activities	2.33 (0.14)	‒2.07 (0.10)	‒0.31 (0.05)	1.62 (0.08)	
3	Lie down	1.34 (0.09)	‒3.37 (0.23)	‒1.67 (0.11)	0.32 (0.06)	
4	Too tired	3.28 (0.23)	‒2.67 (0.14)	‒1.46 (0.06)	‒0.14 (0.04)	1.63 (0.08)
5	Felt fed up or irritated	1.45 (0.09)	‒3.47 (0.23)	‒1.99 (0.12)	‒0.43 (0.06)	1.35 (0.09)
6	Limits concentration	3.52 (0.27)	‒2.72 (0.15)	‒1.59 (0.07)	‒0.31 (0.04)	1.39 (0.06)

*a* slope, *b* thresholds, *HIT-6* 6-item Headache Impact Test, *SE* standard error

### Item characteristics

**Fig. 1:**
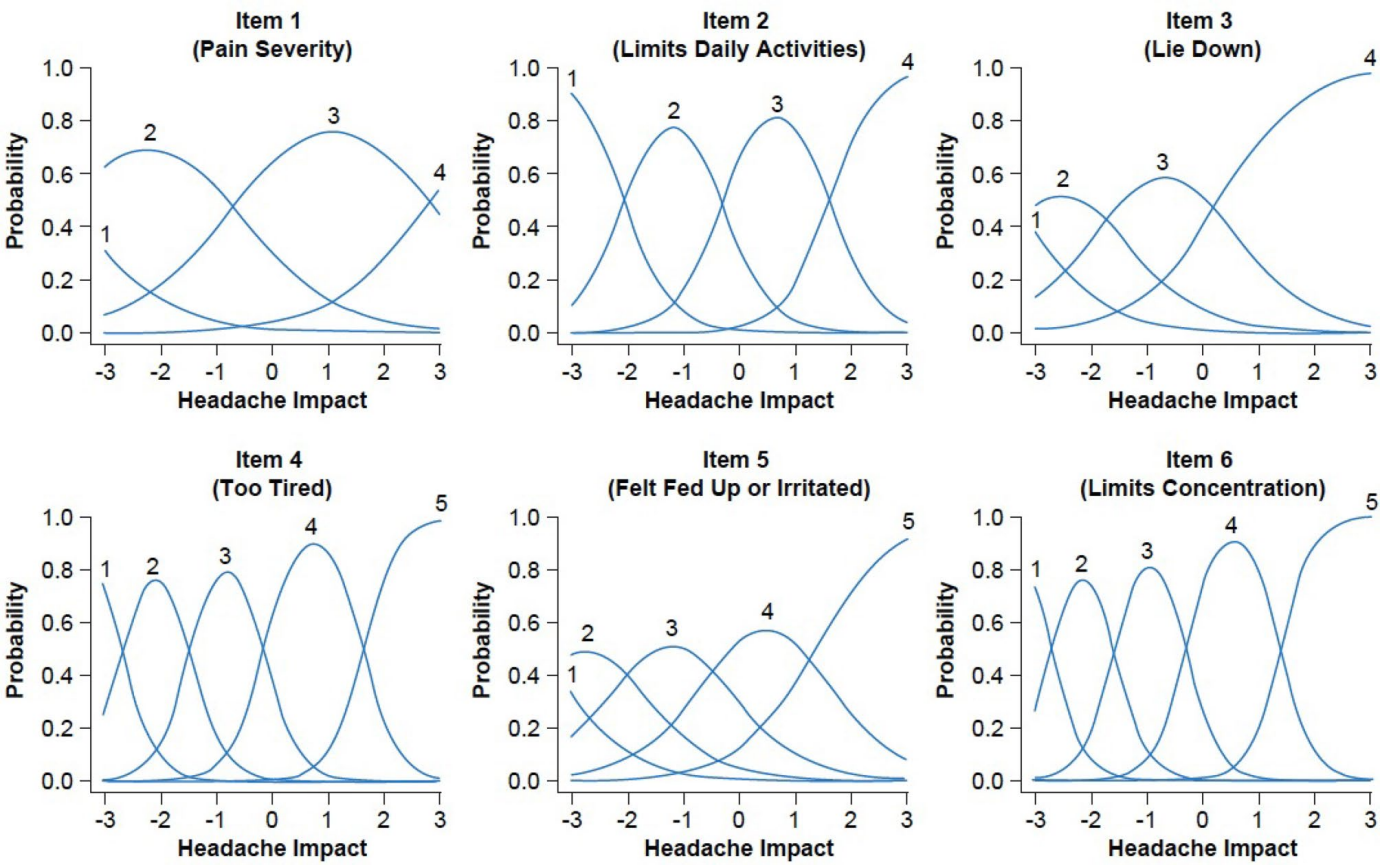
Trace line plots for HIT-6 items using PROMISE-2 baseline data. *HIT-6* 6-item Headache Impact Test

Figure 1 provides graded trace line plots for each HIT-6 item, which depict the relationship between response categories and the severity of the underlying construct of “headache impact”. All six items also demonstrated clearly distinct peaks for most response categories, suggesting that, per item, each response category, as collapsed, was uniquely contributing to the available statistical information (approximately equal to 1/squared SE; [49]). Where less distinct peaks were observed, it was at the lower end of the continuum/thresholds separating the less severe response categories; given the use of baseline data and the severity of the CM patient population, the lack of specificity between less severe response categories was not surprising and is not considered problematic. Additionally in Fig. 1, it can be seen that all HIT-6 items provided an adequate amount of statistical information across a large majority of the continuum of the latent construct of headache impact. The steepness of the curves (see also the a-parameter estimates in Table 4) further suggest that each item contributed reliable information to the total score.

### Reliability

**Fig. 2:**
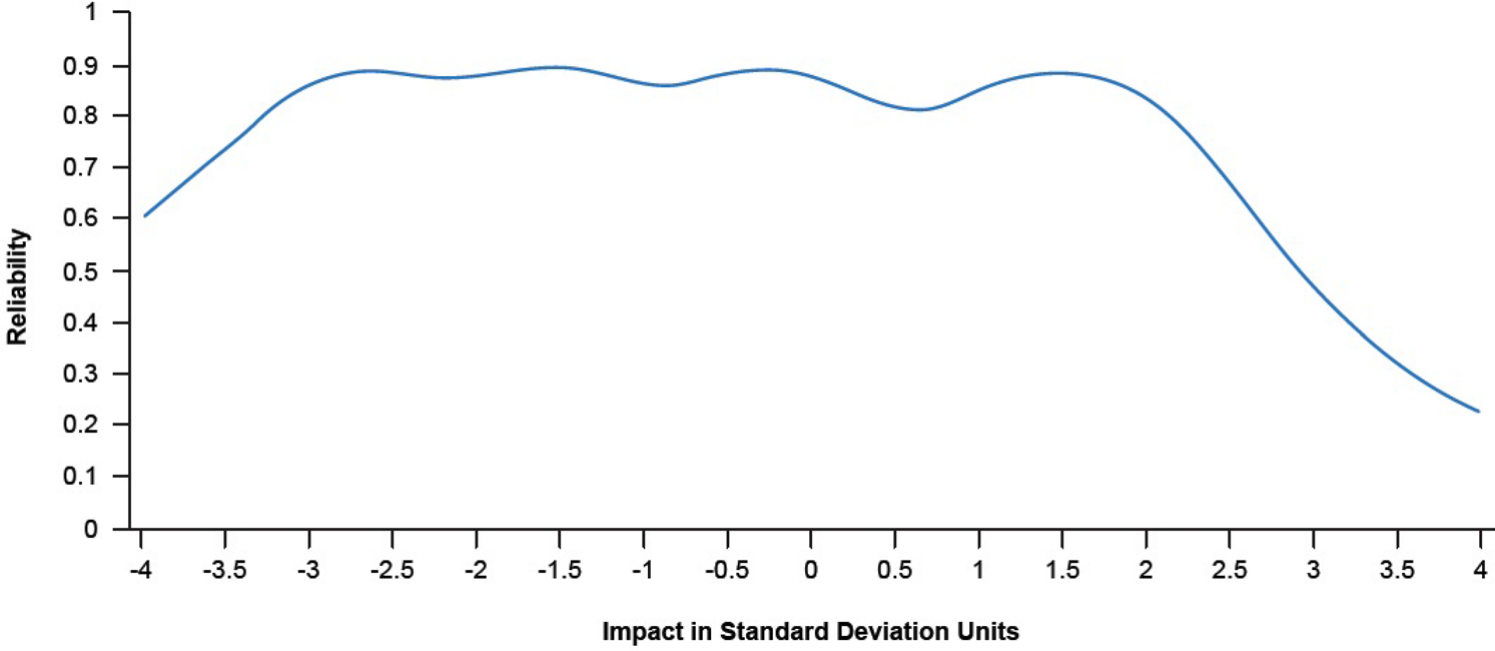
Expected reliability plot of HIT-6 total scores over a range of impact scores. *X*-axis represents impact measured on *z*-score metric (mean = 0, standard deviation = 1). Reliability (*y*-axis) of 0.7 is considered acceptable. *HIT-6* 6-item Headache Impact Test

The reliability curve from the IRT-based evaluation of the HIT-6 (Fig. 2) suggested that the IRT-based HIT-6 total score provided adequate reliability (> 0.70) from approximately − 3.5 to 2.3 standard deviations (SDs) around the mean in this CM sample. The marginal (i.e., distributional) reliability provided by IRT, similar to coefficient alpha, provides a single reliability value. For the HIT-6 overall IRT scores the marginal reliability was 0.86, also well above the specified acceptable minimum value of 0.70.

Coefficient alpha was also found to be above the specified minimum value using both the traditional Pearson approach (α_1_ = 0.82) and the polychoric correlation approach (α_2_ = 0.85) (Table 5). Item-total correlations suggested that the individual items differed in their strength of relationship with the HIT-6 overall summed score, but based on the more appropriate polychoric correlation, each item had a strong relationship with the total score.

**Table 5:** Item response theory alpha and item-total correlations at PROMISE-2 baseline

Item	Item content	Pearson (α_1_)	Polychoric (α_2_)
—	HIT-6 total score	0.82	0.85

		Alpha if item removed	
1	Severe pain	0.82	0.87
2	Limits daily activities	0.78	0.81
3	Lie down	0.81	0.84
4	Too tired	0.76	0.81
5	Felt fed up or irritated	0.80	0.84
6	Limits concentration	0.76	0.80

		Item-total correlation	
		Pearson	Polychoric
1	Severe pain	0.42	0.58
2	Limits daily activities	0.64	0.84
3	Lie down	0.49	0.74
4	Too tired	0.71	0.83
5	Felt fed up or irritated	0.54	0.71
6	Limits concentration	0.72	0.87

*HIT-6* 6-item Headache Impact Test

Test–retest reliability of the HIT-6 total summed score was evaluated between the screening and baseline assessment periods and was found to be approximately 0.67 (Pearson, 0.68; ICC, 0.65), slightly less than the commonly used threshold of 0.70.

### Convergent and Discriminant Correlations

As expected, week 12 correlations among HIT-6 total scores and reference measures were generally greater in magnitude than those at baseline (Table 6). All observed correlations conformed to expectations with respect to direction. The magnitudes of the correlations were generally consistent with expectations, although the observed correlation fell just outside the a priori hypothesized range in several cases. For instance, the magnitude of the correlation for SF-36 emotional role functioning (*r* = − 0.40) was greater than the expected prediction of 0.00 to − 0.30; however, this value is in line with the reported correlation of ‒ 0.37 by Kawata et al. [16]. Additionally, the observed correlation between frequency of MMDs and the HIT-6 total score at baseline, which was lower than anticipated, may be attributable to the fact CM patients needed to have a relatively high migraine frequency at baseline to meet study inclusion criteria. The noticeable increase in the observed correlation from baseline to week 12, in which mean migraine frequency was lower and more variable (Table 3), provides support for this interpretation.

**Table 6:** Convergent and discriminant correlations of the HIT-6 total score with reference measures

Reference measure	Expectation	Baseline	Expectation met at baseline?	Week 12	Expectation met at week 12?
MMDs (past 4 weeks)	0.30 to 0.50	0.19	Direction	0.51	Direction
SF-36 bodily pain	‒0.10 to ‒0.50	‒0.37	Direction & Magnitude	‒0.52	Direction
SF-36 physical role functioning	‒0.30 to ‒0.50	‒0.42	Direction & Magnitude	‒0.56	Direction
SF-36 emotional role functioning	0.00 to ‒0.30	‒0.34	Direction	‒0.40	Direction
EQ-5D-5L usual activities*	0.10 to 0.30	0.29	Direction & Magnitude	0.38	Direction
EQ-5D-5L mobility*	0.00 to 0.10	0.12	Direction	0.14	Direction

*Ordinal variable with Spearman correlation

*EQ-5D-5L* EuroQol five-dimension, five-level scale, *HIT-6* 6-item Headache Impact Test, *MMD* monthly migraine days, *SF-36* Short-Form Health Survey (v2.0)

### Known-groups validity

**Fig. 3:**
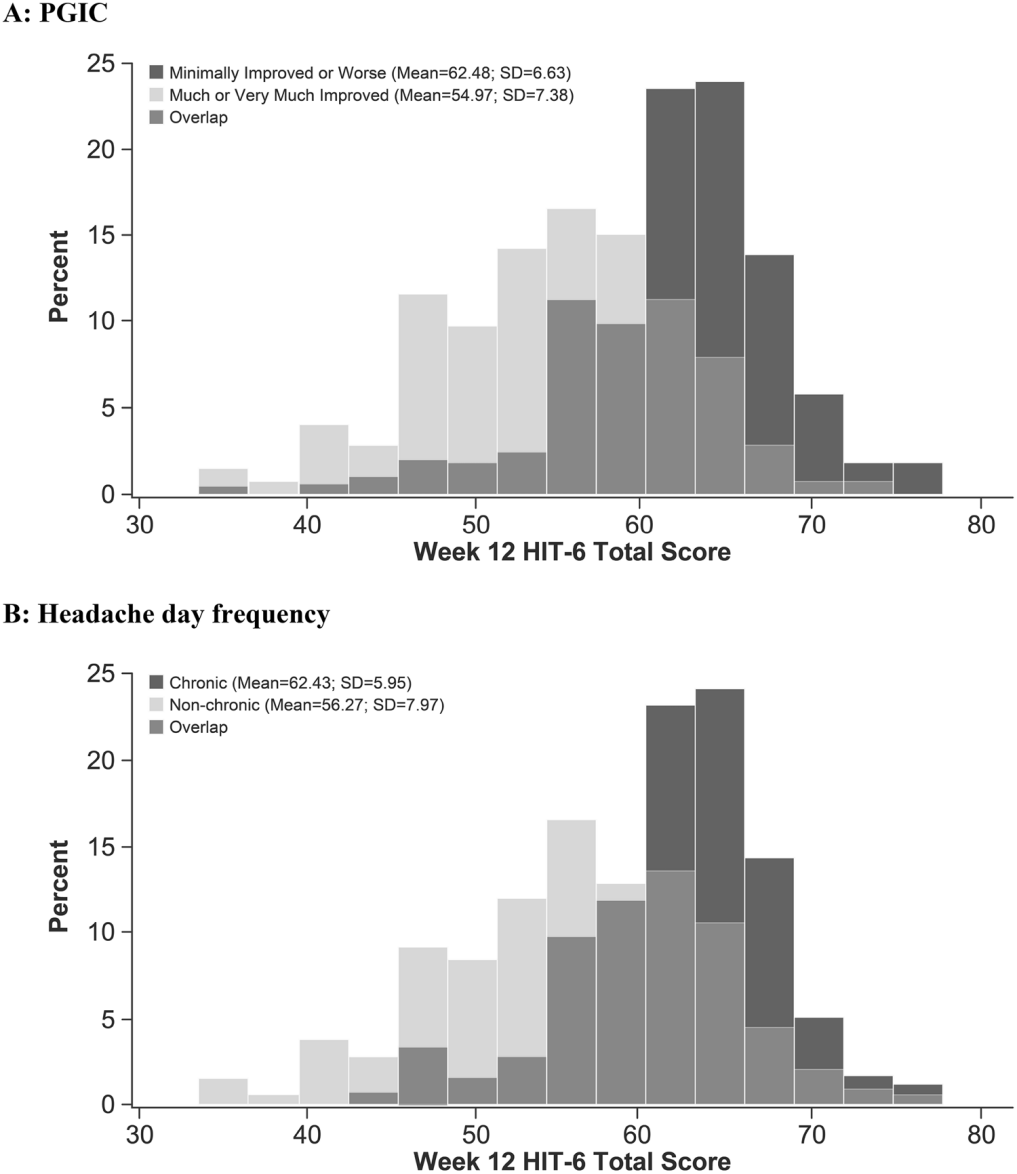
Known-groups analysis of HIT-6 total score. **a** PGIC. **b** Headache day frequency. The chronic subgroup comprises patients with ≥ 15 headache days during weeks 9–12; the non-chronic subgroup comprises patients with < 15 headache days during weeks 9–12. *HIT-6* 6-item Headache Impact Test, *PGIC* Patient Global Impression of Change

HIT-6 total scores conformed to expectations, both in terms of reported mean values and with respect to the outcome of formal tests of difference between the groups (Table 7). Both the improved and non-chronic groups had lower HIT-6 total scores, demonstrating less impact, than the non-improved and chronic headache groups (Fig. 3). The effect size of each difference was large (1.09 and 0.88, respectively), indicating that the HIT-6 total score can distinguish between clinically meaningful groups in the CM population.

**Table 7:** HIT-6 total score by known groups, defined by PGIC and headache day categorizations, at Week 12

Measure	*n*	Mean (SD)
PGIC		
Very much improved or much improved	525	54.97 (7.38)
Minimally improved or worse	498	62.48 (6.33)
Difference		7.50 (6.89)
Cohen’s *d*	1.09	
*p* value	< 0.0001	
Headache days (weeks 9‒12)		
≥ 15 days (“Chronic”)	389	62.43 (5.95)
< 15 days (“non-Chronic”)	635	56.27 (7.97)
Difference		6.15 (7.27)
Cohen’s *d*	0.88	
*p* value	< 0.0001	

*PGIC* Patient Global Impression of Change, *SD* standard deviation

### Sensitivity to change

Correlations between the change in HIT-6 total scores and the change in established reference measures generally conformed to hypothesized directions and magnitudes specified a priori (Table 8). As with the convergent and discriminant correlation results, when outside the expected range, the observed correlations tended to be only slightly larger in magnitude than expected. The primary exception was that the PGIC correlation (*r* = 0.57) was noticeably greater than expected (0.10‒0.30), indicating that change in the HIT-6 total score may be a more robust assessment of general headache impact on patients than initially expected.

**Table 8:** Correlations of HIT-6 total score change scores (baseline to week 12) with reference measures

Reference measure	Expectation	Correlation	Expectation met?
MMDs (past 4 weeks)	0.30 to 0.50	0.48	Direction and Magnitude
Patient global impression of change	0.10 to 0.30	0.57	Direction
SF-36 bodily pain	‒0.50 to ‒0.10	‒0.47	Direction and Magnitude
SF-36 physical role functioning	‒0.50 to ‒0.30	‒0.49	Direction and Magnitude
SF-36 emotional role functioning	‒0.30 to 0.00	‒0.35	Direction
EQ-5D-5L usual activities*	0.10 to 0.30	0.20	Direction and Magnitude
EQ-5D-5L mobility*	0.00 to 0.10	0.12	Direction

*EQ-5D-5L* EuroQol five-dimension, five-level scale, *HIT-6* 6-item Headache Impact Test, *MMDs* monthly migraine days, *SF-36* Short-Form Health Survey (v2.0)

*Denotes categorical item and Spearman correlations

## Discussion

The HIT-6 appears to be a reliable and valid instrument for the assessment of headache impact in patients with CM, based on our analyses using data from PROMISE-2. Although CM shares features with other headache diagnoses, it is important to recognize that these are distinct conditions and, as such, it is critical that headache PROMs are rigorously evaluated for specific use in the CM population. One goal of the current study was to provide a unique psychometric evaluation of the HIT-6 using IRT in a CM sample, and results demonstrated that the HIT-6 was successfully calibrated using a unidimensional IRT model. Correlations of HIT-6 total scores with the reference measures, both cross-sectionally and using longitudinal change scores, conformed to expectations with respect to direction and often conformed to expectations of magnitude. Known-groups analyses and correlation of change scores also supported the contention that the HIT-6 total scores behave in a manner consistent with the assessment of headache impact.

The IRT results demonstrated that all HIT-6 items provided good coverage over the latent construct of headache impact, and each provided valuable information to the total score. These results are similar to a previous study of 1384 patients with CM in which a unidimensional model fitted to the data met the typical cut-values for good fit [19]. Conversely, in a psychometric examination of the HIT-6 in headache clinic patients (*N* = 309) [16], while most items could differentiate between a wide range of individuals with migraine, there was a lack of unique information provided by the lower response categories for the pain severity and wishing to lie down items, suggesting that these items were unable to separate fine-grained differences. Given the severity of migraine for those living with CM, having less information at the lower end of the headache impact continuum should not be problematic in most settings. However, if one expects large, meaningful, positive changes, it may be worth taking advantage of the full HIT item bank using a computerized adaptive administration to maximize measurement precision and reliability over the range of experience.

Internal consistency estimates demonstrated reliable scores across a wide range of headache impact, with good marginal reliability. Moreover, coefficient alpha estimates were also in the acceptable range, and these results were in line with previous examinations of the reliability of the HIT-6 [12, 16–19]. Test–retest reliability between screening and baseline was slightly lower than would be considered acceptable for continued use. However, this is likely due to the homogeneity of the patient sample prior to treatment due to the trial enrollment criteria; limited variability can artificially reduce/attenuate estimates of correlations [46]. Previously reported test–retest values of the HIT-6 scores, despite differences in methodologies and time points used across studies, were found to be at acceptable levels [13, 19].

The results of the convergent/discriminant correlation analyses were largely in line with the previous literature. When correlations did not conform to expectations, the observed correlation value typically fell only slightly outside the expected range and indicated a stronger association than anticipated; review of the original predictions suggests that relationships may have been underestimated given the CM population. The validity of the HIT-6 scores was also supported in the form of convergent and discriminant validity analyses during its initial validation using on online sample of adults (18–65) that self-reported a headache in the past four weeks not due to illness, injury or hangover [13], where, as expected, HIT-6 scores correlated negatively with all subscale and component scores of the 8-item short-form health survey (SF-8), with magnitudes ranging from small to moderate. HIT-6 summed scores also correlated strongly and positively with scores from an adaptive administration of HIT items and IRT-based scores derived from 34 items of the HIT item bank [13]. Subsequent studies using a variety of headache patient samples found HIT-6 total scores to be associated, as expected, with numerous other migraine-specific PROM scores as well as with general health and HRQoL measures and with objective headache and migraine outcomes [12, 13, 17, 19, 45, 50–53].

Results of the known-groups analyses supported the validity of the HIT-6 total scores, in line with previous evaluations [5, 12, 13, 15, 19]. In the initial HIT-6 publication, individuals reporting more severe pain generally demonstrated significantly higher HIT-6 total scores [13]. Other studies have indicated that mean HIT-6 scores significantly increased according to headache diagnosis (non-migraine < EM < CM) [5, 12]. Moreover, in agreement with our data, previous publications have reported that HIT-6 total scores show sensitivity to change in patients with migraine [45, 54–57]. In a clinical trial investigating erenumab injections in patients with EM, active treatment reduced mean MMDs and days with acute migraine medication use relative to placebo [58]; HIT-6 total score data mirrored these results, with the treatment groups demonstrating statistically larger decreases from baseline relative to placebo [54]. In the PREEMPT clinical trials of onabotulinumtoxinA in patients with CM [55–57], the HIT-6 was employed as a secondary outcome and demonstrated a statistically significant reduction in mean scores from baseline to week 24, favoring active treatment. In the same study’s open-label phase (in which previously placebo-treated patients received active treatment), the HIT-6 total scores retained the demonstrated decrease from baseline but the differences between treatment groups were no longer statistically significant, as would be expected.

The HIT-6 appears to be a valuable tool for measuring headache impact in patients with CM in a clinical setting, and additional studies are warranted to empirically evaluate and develop threshold(s) for clinically meaningful change (responder definitions) in individuals with CM to help facilitate clinical decision making. Psychometric analyses should also be undertaken to test whether the measurement properties of the HIT-6 are equivalent across different headache groups, such as EM. Although the current study had several strengths—including the large sample size, rigorous psychometric modeling, evaluation of item characteristics, and assessment of reliability—there were limitations as well. The most notable is that the data were from a clinical trial, and thus comprised a more homogeneous sample than the general patient population due to enrollment criteria, potentially limiting the generalizability of the data. The impact of this homogeneity was evident in the screening and baseline HIT-6 scores that resulted in what were likely attenuated estimates of test–retest reliability; we recommend that this be re-examined in a prospective observational study to examine the accuracy of this supposition and provide a more complete understanding of the psychometric soundness of the HIT-6 in the CM population.

## Conclusion

This body of work examined the reliability and validity of the HIT-6 in patients with CM using data from the large PROMISE-2 clinical trial. All HIT-6 items provided coverage over the range of headache and migraine impact, as well as unique and reliable information to the total score, and the validity of the HIT-6 for measuring impact of headache on daily life in individuals with CM was supported. The short administration time, easy scoring, and interpretability of the HIT-6 make it an excellent tool for use in applied research, clinical trials, and clinical practice settings so that broader patient experience can be assessed.
